# Mechanism of Choline Chloride in Alleviating Hepatic Lipid Deposition in Bighead Carp (*Aristichthys nobilis*): Insights from Glycerophospholipid Metabolism and Choline Metabolic Pathways

**DOI:** 10.3390/ani16152345

**Published:** 2026-08-01

**Authors:** Huimin Sun, Jun Chen, Chengjie Wang, Feng Huang, Meiqin Zhuo

**Affiliations:** Hubei Key Laboratory of Animal Nutrition and Feed Science, School of Animal Science and Nutritional Engineering, Wuhan Polytechnic University, Wuhan 430023, China; sunhuimin020719@163.com (H.S.); hychenjun@163.com (J.C.); 19935057223@163.com (C.W.); huangfeng0001@aliyun.com (F.H.)

**Keywords:** choline, bighead carp, lipid metabolism, choline metabolism, lipidomics

## Abstract

This study integrated an in vivo feeding trial (0.00–1.00% choline chloride) with in vitro primary hepatocyte experiments to elucidate the molecular mechanisms by which choline chloride alleviates hepatic lipid deposition in bighead carp. The results showed that dietary supplementation with 0.80–1.00% choline chloride significantly reduced hepatic triglyceride (TG) content and lipid droplet accumulation. At the molecular level, choline markedly downregulated lipogenic genes (*g6pd*, *accα*, *fas*) while upregulating genes involved in lipolysis (*cpt1α*, *acox1*) and lipid transport (*apob-100*, *mttp*). Non-targeted lipidomics revealed that choline maintains lipid homeostasis primarily via the glycerophospholipid metabolism pathway, significantly increasing levels of phosphatidylcholine (PC), phosphatidylethanolamine (PE), and phosphatidylserine (PS). Furthermore, choline intervention enhanced the expression of its own metabolism-related genes (*slc44a2*, *chdh*, *ck*, *chpt1*), accelerating choline transport and phosphorylation. Overall, based on the results of this experiment, 0.80% is the recommended supplemental level of choline chloride in diets, which can effectively alleviate lipid deposition in bighead carp. The mechanism involves inhibiting lipogenesis and promoting lipid decomposition and transport through glycerophospholipid pathways, while simultaneously enhancing PC synthesis to accelerate hepatic lipid export. These findings provide a theoretical basis for precise feed formulation in bighead carp.

## Simple Summary

This study combined in vivo feeding trials with dietary choline levels ranging from 0.00% to 1.00% and in vitro primary hepatocyte experiments to explore the molecular mechanisms by which choline chloride alleviates hepatic lipid deposition in bighead carp. We detected alterations in liver histomorphology, hepatic lipid accumulation, as well as the activities and gene expression of key lipid-metabolizing enzymes. The overall results indicated that dietary supplementation with 0.80% choline chloride could effectively prevent hepatic lipid deposition in bighead carp. The underlying mechanism is as follows: it inhibits lipogenesis and promotes lipolysis and lipid transport via the glycerophospholipid metabolism pathway, while improving phosphatidylcholine synthesis to accelerate hepatic lipid export. The findings provide a theoretical basis for the precise feed formulation of bighead carp.

## 1. Introduction

Choline is an essential water-soluble vitamin for animal growth and development. It can promote the oxidative utilization of fatty acids in fish liver, reduce excessive lipid deposition, and improve hepatic health and growth performance of fish [1,2]. Lipid deposition in fish results from the balance among lipogenesis, fatty acid β-oxidation, and lipid transport [3]. Fatty acid synthase (FAS) and acetyl-CoA carboxylase (ACC) play crucial roles in lipogenesis. Carnitine palmitoyl transferase 1α (CPT1α) and acyl-CoA oxidase (ACOX) are involved in fatty acid β-oxidation. Microsomal triglyceride transfer protein (MTTP) is an essential lipid transfer protein required for the synthesis, assembly, and secretion of very low-density lipoprotein (VLDL) [4]. Previous studies have demonstrated that choline can regulate the expression of these genes to modulate lipid metabolism, thereby reducing hepatic lipid deposition in fish [5,6]. Supplementing 10 g/kg and 15 g/kg choline chloride into high-lipid diets for hybrid grouper (♀ *Epinephelus fuscoguttatus ×* ♂ *E. lanceolatus*) resulted in the minimum levels of hepatic triglycerides and total cholesterol [7]. Pacific white shrimp (*Litopenaeus vannamei*) fed the diet containing 4.67 g/kg choline had the highest values of related hepatopancreatic indicators and mRNA expression of *fas*, *srebp,* and *acc*, which were significantly higher than those in the lowest choline group [8].

On the other hand, choline can be oxidized to betaine in the animal organism. Betaine improves the oxidation process of fatty acids in cellular mitochondria, elevates the content of long-chain acylcarnitines in the liver, promotes lipolysis, and reduces lipid deposition in the liver and somatic tissue [9]. Choline is phosphorylated under the catalysis of choline kinase (CK) and generates phosphatidylcholine (PC) via the CDP-choline pathway. As one of the major components of VLDL, PC enables choline to indirectly participate in lipid metabolism and transport in vivo. It promotes hepatic utilization of fatty acids, accelerates the efflux of hepatic TG, and prevents the formation of fatty liver [10]. Studies have shown that adequate dietary choline can upregulate the mRNA expression level of *mttp*, enhance the assembly and secretion of VLDL, and facilitate the outward transport of hepatic lipids [11].

Lipids exert multiple essential roles in cellular functions. As fundamental components of biological membranes and important metabolites of organisms, lipids play pivotal roles in cellular energy storage, cell barrier formation, membrane matrix construction, and signal transduction [12,13]. Lipidomics has emerged as an important analytical technique in fish research. In the field of environmental toxicology, it is widely applied to elucidate the patterns of lipid metabolism disorders in fish under pollutant stress [14]. In aquaculture nutrition, lipidomics acts as a core tool to decipher the mechanisms of lipid metabolism in fish. It can systematically characterize the effects of dietary nutrients on in vivo lipid profiles, precisely detect compositional changes in hepatic lipid molecules including triglycerides and glycerophospholipids, and identify the underlying metabolic regulatory pathways [15,16,17]. At present, research regarding the impacts of choline on lipid metabolism in fish remains scarce. For this reason, the present work concentrated on hepatic lipid metabolism to clarify the molecular mechanism underlying the alleviation of hepatic lipid deposition by choline chloride in bighead carp.

Bighead carp (*Aristichthys nobilis*) is one of the four major Chinese carps, which belongs to filter-feeding freshwater fish. It is also known as fathead carp, characterized by a laterally compressed body and a large head. This species mainly feeds on zooplankton and exhibits a fast growth rate [18]. As a core cultured freshwater fish species, it is widely cultivated nationwide mostly under polyculture systems with huge market demand. However, excessive hepatic lipid deposition frequently occurs during culture, which restricts the improvement of breeding efficiency and product quality [19]. According to the 2025 *China Fishery Statistical Yearbook* [20], the aquaculture production of bighead carp reached 3.49 million tons in 2024. However, little attention has been paid to the nutritional requirements of bighead carp by other laboratories both domestically and internationally. Our laboratory has previously investigated the dietary requirements of protein [21], lipid [22], phosphorus [23,24], calcium [25,26], and iron in bighead carp [27]. Nevertheless, the regulatory mechanism of choline on lipid metabolism in this species remains unclear. In this study, we explored the intrinsic mechanism by which choline reduces hepatic lipid deposition in bighead carp through in vivo and in vitro experiments combined with lipid metabolomics, aiming to provide theoretical evidence for understanding the pathogenesis and developing prevention strategies of fatty liver in farmed bighead carp.

## 2. Materials and Methods

### 2.1. Animal Ethics

All animal experimental protocols were reviewed and approved by the Animal Care and Use Committee of Wuhan Polytechnic University (Approval No. WPU202207003), Wuhan, Hubei, China.

### 2.2. Feeding Trial

#### 2.2.1. Diet Formulation Design

In this trial, soybean meal and fish meal were used as the main protein sources, soybean oil and corn oil (mixed at a ratio of 1:1) as the lipid sources, and starch and other raw materials were supplemented. The endogenous choline content of the basal diet was consistent across all groups. All raw materials were purchased from Wuhan Aohua Agriculture & Animal Husbandry Co., Ltd. (Wuhan, China). Choline chloride (50% purity) was applied as the choline source to prepare six isonitrogenous experimental diets. The supplemental levels of choline were set at 0.00%, 0.20%, 0.40%, 0.60%, 0.80% and 1.00%, which were equivalent to 0.00, 2.00, 4.00, 6.00, 8.00 and 10.00 g per kilogram of diet, respectively. Cellulose served as an inert filler and was proportionally reduced as choline chloride levels increased to maintain equal nitrogen and energy contents across all experimental diets. All dietary ingredients of each group were thoroughly blended stepwise, mixed with water, and pelleted using a pellet mill. After natural air-drying, the diets were ground and sieved through a 40-mesh sieve (0.63 mm), then stored in a refrigerator at −20 °C for subsequent use. The formulation and proximate composition of experimental diets are presented in Table 1. Crude protein in diets was determined according to GB/T 6432-2018 [28], crude fat according to GB/T 6433-2025 [29], crude ash content according to GB/T 6438-2025 [30], and moisture content according to GB/T 6435-2014 [31].

#### 2.2.2. Experimental Design of Feeding Trial

The feeding experimental design in this study referred to the method described by [32]. Juvenile bighead carp were purchased from Xingfucun Fishery, Huanggang City, Hubei Province, and transported to the Laboratory of Fish Nutrition and Metabolism, Wuhan Polytechnic University, Hubei Province. The experimental fish were disinfected with povidone-iodine and then reared in temporary culture ponds for a 2-week acclimation period. A total of 900 healthy, uniform-sized juvenile bighead carp with an initial body weight of 1.77 ± 0.20 g were randomly selected. The fish were randomly allocated into 6 groups with 3 replicate tanks per group. Each tank (60 cm diameter × 120 cm height) held 50 fish with an effective water volume of 300 L. The six formulated experimental diets were randomly assigned to the 18 aquariums, with three replicates per dietary treatment. During the experiment, fish were fed to apparent satiation twice a day at 9:00 and 17:00. Residual feed and feces were siphoned daily to maintain water quality. The feeding trial lasted for 66 days, with the water temperature maintained at 26–28 °C and continuous aeration provided for 24 h per day. Water quality parameters were measured once weekly. During the experiment, the main water quality indicators were as follows: total ammonia nitrogen <0.05 mg/L, nitrite nitrogen <0.01 mg/L. After the feeding trial, all fish were fasted for 24 h before sampling. Fish were dissected on ice to isolate liver tissues. Samples intended for histological sectioning were immersed in tissue fixative and stored at room temperature. Samples used for the determination of TG content and gene expression were divided into cryovials, snap-frozen in liquid nitrogen, and subsequently preserved in an ultra-low temperature refrigerator at −80 °C.

#### 2.2.3. Histological Observation of Liver Tissue

Hematoxylin-eosin staining: Liver tissues were fixed in 4% paraformaldehyde (Sinopharm Chemical Reagent Co., Ltd., Shanghai, China) for 24 h, then subjected to serial ethanol (Sinopharm Chemical Reagent Co., Ltd., Shanghai, China) dehydration, xylene (Sinopharm Chemical Reagent Co., Ltd., Shanghai, China) clearing, and paraffin embedding. The tissues were sectioned into 4 μm slices and baked in an oven at 60 °C. Subsequently, the sections were stained with hematoxylin and eosin (H&E), then observed and photographed under an optical microscope (Ningbo Sunny Instruments Co., Ltd., Ningbo, China).

Oil Red O staining: Liver tissues were fixed in 4% paraformaldehyde (Sinopharm Chemical Reagent Co., Ltd., Shanghai, China) solution for 24 h, embedded with OCT compound, and sectioned using a cryostat. The sections were fixed in tissue fixative for 15 min and rinsed with tap water. Subsequently, the sections were stained with Oil Red O staining solution, differentiated with 60% isopropanol (Sinopharm Chemical Reagent Co., Ltd., Shanghai, China), and rinsed with pure water. Then the sections were counterstained with hematoxylin (Sinopharm Chemical Reagent Co., Ltd., Shanghai, China) and washed again with pure water, followed by differentiation in 1% hydrochloric acid (Sinopharm Chemical Reagent Co., Ltd., Shanghai, China) and rinsing with distilled water. Sections were blued in 0.6% ammonia water (Sinopharm Chemical Reagent Co., Ltd., Shanghai, China) and rinsed with tap water. After microscopic examination of staining quality, the slides were mounted with glycerol gelatin, and observed and photographed under an optical microscope (Ningbo Sunny Instruments Co., Ltd., Ningbo, China).

#### 2.2.4. Untargeted Lipid Metabolomics Analysis of Liver

Liver samples collected from the 0.00% choline (control) and 0.80% choline supplementation groups were sent to Shanghai Majorbio Bio-Pharm Technology Co., Ltd., Shanghai, China, for untargeted lipidomic profiling. The liver tissues were ground into powder in liquid nitrogen. Lipids were extracted using a methanol-chloroform mixture (2:1, *v*/*v*). After vortexing and ultrasonication, the mixture was centrifuged at 12,000 r/min for 15 min. The lower organic phase was collected, dried under nitrogen flow, re-dissolved with methanol, and filtered through a 0.22 μm membrane. The prepared solution was then transferred into sample vials for subsequent liquid chromatography-tandem mass spectrometry (LC-MS/MS) analysis. Raw data were imported into LipidSearch (Thermo, San Diego, CA, USA) for baseline filtering, peak identification, peak integration, retention time correction, peak alignment and lipid annotation. A final data matrix containing lipid identities, retention times, mass-to-charge ratios and peak intensities was generated. The resulting data matrix was further analyzed on the Majorbio Cloud Platform, including total lipid classification, screening of differential lipid metabolites, and KEGG pathway enrichment analysis of differential lipids. Differential lipid metabolites between the 0% and 0.80% groups were screened using the following criteria: VIP > 1, |log_2_(Fold Change)| > 1, and *p* < 0.05. The analytical procedure in this study referred to the previously published method of our research group [33].

### 2.3. Cell Experiment

#### 2.3.1. Isolation and Culture of Primary Hepatocytes from Bighead Carp

Experimental fish were exsanguinated via gill arch cutting, and approximately 15 g of liver tissue was dissected and minced. The fish body was disinfected with 75% alcohol (Sinopharm Chemical Reagent Co., Ltd., Shanghai, China) and transferred to a biosafety cabinet. The liver was dissected out and rinsed twice with PBS containing antibiotics (Shanghai Macklin Biochemical Co., Ltd., Shanghai, China), then placed in a Petri dish filled with transition medium and minced into pieces. After discarding the medium, 0.25% trypsin (Shanghai Macklin Biochemical Co., Ltd., Shanghai, China) was added for digestion for 5 min, followed by neutralization medium to terminate digestion. The cell suspension was collected, and the digestion–neutralization–collection procedure was repeated 5–6 times. The collected cell suspension was sequentially filtered through 100 μm and 70 μm cell strainers. The filtrate was centrifuged at 1000 r/min (Xiangyi Centrifuge Instrument Co., Ltd., Changsha, China) for 5 min at room temperature to obtain cell pellets. After centrifugation, the supernatant was discarded, and the cell pellets were resuspended in maintenance medium and cultured in an incubator at 28 °C. The isolated suspended hepatocytes were stained with 0.5% trypan blue (Sinopharm Chemical Reagent Co., Ltd., Shanghai, China). Cells with a viability higher than 95% were used for subsequent experiments.

#### 2.3.2. Establishment of High-Lipid Hepatocyte Model

An in vitro high-lipid hepatocyte model was established by incubating bighead carp hepatocytes with oleic acid (OA) (Sinopharm Chemical Reagent Co., Ltd., Shanghai, China). Referring to the method described by Du et al. [34], primary hepatocytes were incubated with OA at concentrations of 0.0, 0.1, 0.2, 0.4, and 0.8 mmol/L for 48 h. Cell viability was determined by the MTT assay, and cellular TG content was measured using commercial kits purchased from Nanjing Jiancheng Bioengineering Institute, Nanjing, China. The optimal OA concentration was determined to be 0.2 mmol/L based on a significant increase in intracellular TG content without adverse effects on cell viability.

#### 2.3.3. Determination of Choline Treatment Concentration

Six choline concentrations of 0, 25, 50, 75, 100, and 150 μmol/L were set and co-incubated with hepatocytes together with 0.2 mmol/L OA for 48 h. Cell viability and intracellular TG content were determined. The optimal choline concentration of 50 μmol/L was selected, which significantly reduced lipid deposition without exhibiting cytotoxicity to hepatocytes. Therefore, in the subsequent cell experiments, we established three treatment groups: Control group, 0.2 mmol/L OA (OA group), and 0.2 mmol/L OA + 50 μmol/L choline chloride (OA + CC) group.

### 2.4. Determination of TG Content and FAS Activity

The contents of triglyceride (TG) and total protein (TP) were determined using kits from Nanjing Jiancheng Bioengineering Institute, Nanjing, China. (Cat. No.: A110-1-1, GPO-PAP enzymatic method; Cat. No.: A045-2-2, Coomassie brilliant blue method). The fatty acid synthase (FAS) assay kit was purchased from Shanghai Enzyme-linked Biotechnology, Shanghai, China (Cat. No.: ml076651). All determinations were performed strictly in accordance with the manufacturer’s instructions.

### 2.5. Total RNA Extraction and Quantitative Real-Time PCR (qRT-PCR)

Total RNA was extracted from liver tissues and hepatocytes following the manufacturer’s protocol of RNAiso Plus (9109, TaKaRa, Kyoto, Japan). Reverse transcription was performed using the PrimeScript™ RT reagent Kit with gDNA Eraser (RR047A, Takara Bio Inc., Shiga, Japan). RT-qPCR detection was carried out with TB Green^®^ Premix Ex Taq™ (Tli RNaseH Plus) (RR420A, Takara Bio Inc., Shiga, Japan) according to the experimental method described by [35]. The relative mRNA expression levels of target genes were calculated using the 2^−ΔΔCt^ method, with *β-actin* and *gapdh* serving as dual reference genes. The geometric mean of the Ct values of the two reference genes was used for normalization [35]. All primer sequences are listed in Table 2.

### 2.6. Statistical Analysis of Data

Normality test and homogeneity of variance test were performed prior to data analysis. All statistical analyses were carried out using SPSS 20.0. One-way analysis of variance (ANOVA) combined with Duncan’s multiple range test was applied to compare significant differences among groups with choline substitution levels ranging from 0.00% to 1.00%. A difference at *p* < 0.05 was considered statistically significant. Different lowercase letters indicated significant differences between groups with varying dietary choline levels (*p* < 0.05). An unpaired t-test was used to analyze data between the 0.00% and 0.80% choline supplementation groups. All figures were generated using GraphPad Prism 8.0. Asterisk notations represent significance levels: * for *p* < 0.05, ** for *p* < 0.01, and *** for *p* < 0.001. A total of six treatment groups were designed, with three replicates per group. One fish was sampled from each replicate (*n* = 3).

## 3. Results

### 3.1. Effects of Dietary Choline Levels on Histological Structure, Lipid Deposition and Expression of Lipid Metabolism-Related Genes in the Hepatic Tissue of Bighead Carp

**Figure 1:**
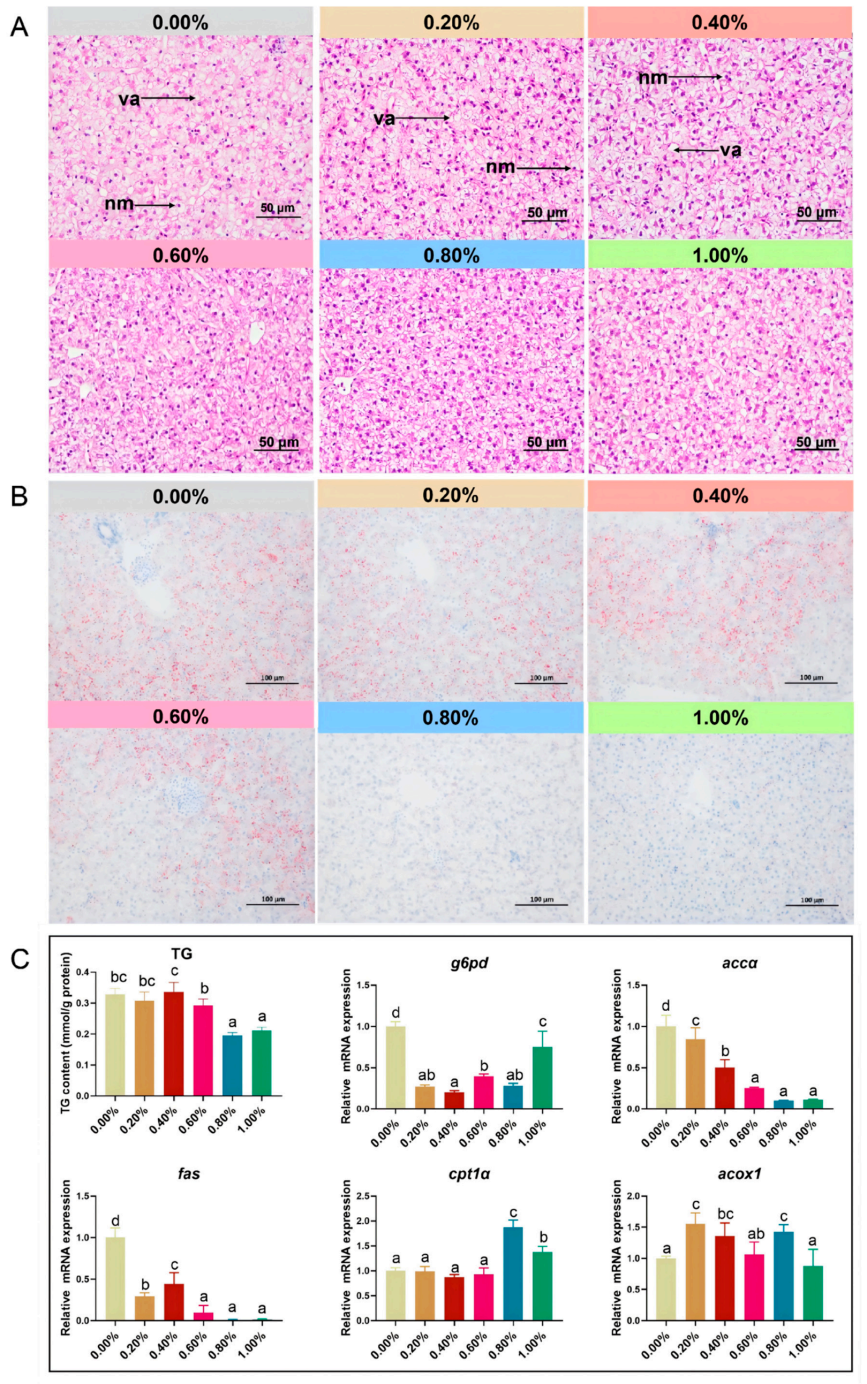
Effects of dietary choline levels on histological structure, lipid deposition and expression of lipid metabolism-related genes in the liver of bighead carp. (**A**): H&E staining (nm: nuclear misalignment, va: vacuole); (**B**): Oil Red O staining (red: lipid droplets, blue: nucleus); (**C**): Hepatic TG content and expression levels of lipid metabolism-related genes. Data were expressed as means with SD (*n* = 3). Distinct superscripts above the bars indicate significant variations (*p* < 0.05). Note: Results were expressed as mean ± standard deviation (SD) (*n* = 3).

H&E staining results showed that hepatocytes in the 0.00%, 0.20% and 0.40% choline supplementation groups exhibited obvious vacuolization and nuclear displacement, while the vacuolation and nuclear displacement of hepatocytes were significantly alleviated in the 0.60%, 0.80% and 1.00% choline supplementation groups (Figure 1A). Oil Red O staining revealed massive lipid droplet accumulation in the liver of the control group and low-dose choline groups, while the number of lipid droplets was significantly reduced in the 0.80% and 1.00% choline supplementation groups (Figure 1B).

The results of hepatic TG content and gene expression showed that compared with the control group, hepatic TG content was significantly decreased in the 0.80% and 1.00% choline supplementation groups (*p* < 0.05). The expression levels of lipogenic genes *accα* and *fas* were significantly down-regulated with the increase in dietary choline level (*p* < 0.05). The expression of the lipolytic gene *cpt1α* reached the highest level in the 0.80% supplementation group, and the expression of *acox1* was significantly up-regulated in the 0.20%, 0.40% and 0.80% supplementation groups (*p* < 0.05) (Figure 1C).

### 3.2. PCA and OPLS-DA Analyses of the Hepatic Lipidome

**Figure 2:**
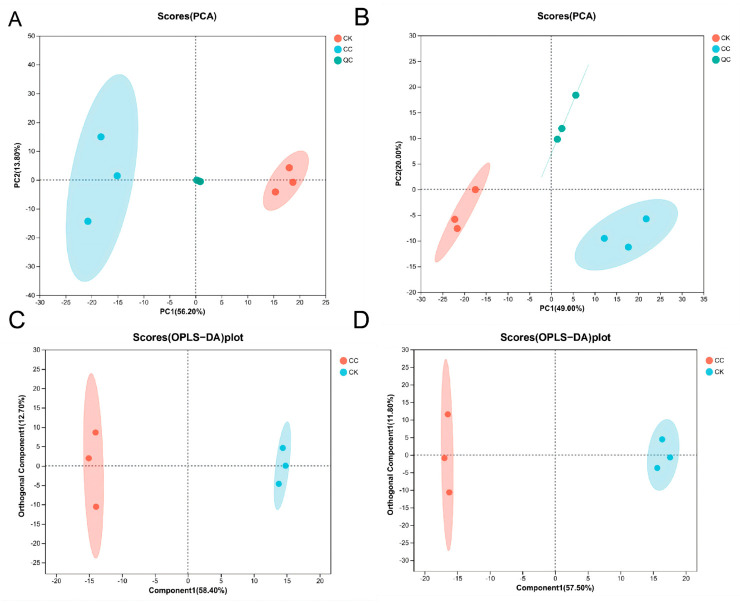
Statistical analysis of hepatic lipidome between the control group and 0.80% choline group. (**A**): PCA score plot in positive ion mode; (**B**): PCA score plot in negative ion mode; (**C**): OPLS-DA score plot in positive ion mode; (**D**): OPLS-DA score plot in negative ion mode.

Based on the previous results, the 0.00% (control group) and 0.80% (choline supplementation group) were selected for lipidomics analysis and detection of the expression levels of genes involved in choline metabolism. PCA and OPLS-DA analyses showed that under positive and negative ion modes, samples in the control and choline groups exhibited tight intra-group aggregation and distinct inter-group separation. Quality control samples were densely distributed, indicating good detection stability. In positive ion mode, R^2^X = 0.813 and Q^2^ = 0.964; in negative ion mode, R^2^X = 0.809 and Q^2^ = 0.948. The established model presented excellent fitness and predictive ability (Figure 2).

### 3.3. Effects of Dietary Choline Levels on the Untargeted Hepatic Lipidome and Choline Metabolism-Related Gene Expression in Bighead Carp

**Figure 3:**
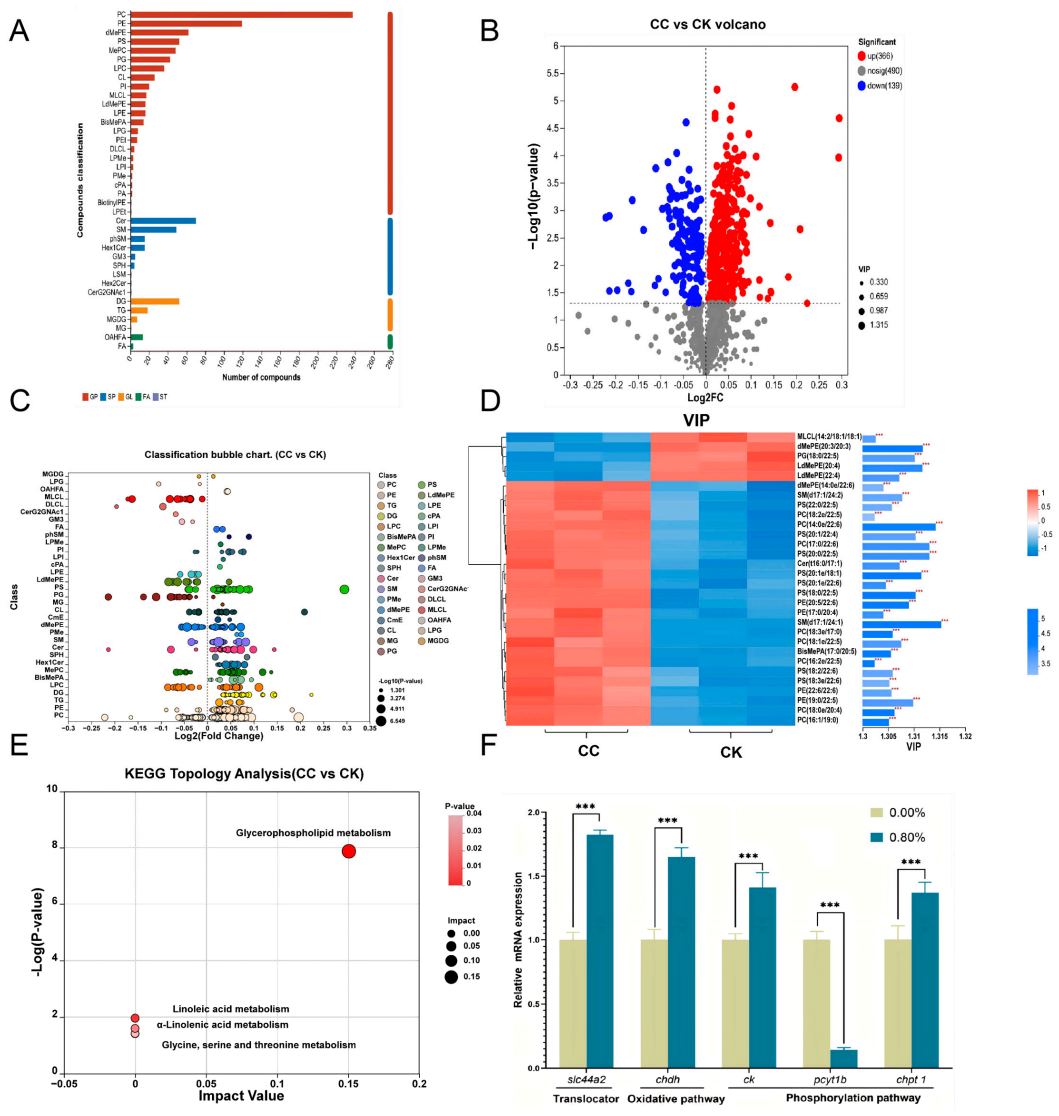
Analysis of differential lipid metabolites and choline metabolism-related gene expression in the liver between the control group and 0.80% choline group. (**A**): Classification statistics of lipid molecules; (**B**): Volcano plot of differential lipids; (**C**): Bubble plot of differential lipid molecules; (**D**): Heatmap of differential metabolites; (**E**): KEGG pathway enrichment analysis; (**F**): Expression of choline metabolism-related genes. Note: *** *p* < 0.001. Results were expressed as mean ± standard deviation (SD) (*n* = 3).

A total of 995 lipid molecules were identified in this study, including 738 glycerophospholipids, 161 sphingolipids, 78 glycerides, 16 fatty acyls, and two sterol lipids (Figure 3A). Using *p* < 0.05 as the screening threshold, we identified 505 differentially expressed lipid metabolites, among which 366 were significantly upregulated (predominantly PC, PE, and PS) and 139 were downregulated (mainly PG and MLCL) (Figure 3B,C). The heatmap of the top 30 differential metabolites ranked by VIP value showed that lipid molecules including PC (18:2e/22:5), PC (14:0e/22:6), PC (17:0/22:6), PS (20:1/22:4), PE (20:5/22:6), SM (d17:1/24:1), and Cer (t16:0/17:1) were markedly accumulated in the choline group. In contrast, MLCL (14:2/18:1/18:1), dMePE (20:3/20:3), PG (18:0/22:5), LdMePE (20:4), and LdMePE (22:4) were significantly decreased (Figure 3D). KEGG pathway enrichment analysis identified four significantly enriched pathways, including glycerophospholipid metabolism, linoleic acid metabolism, α-linolenic acid metabolism, and glycine, serine, and threonine metabolism, among which glycerophospholipid metabolism served as the core pathway (Figure 3E). Detection of choline metabolism-related genes indicated that the expression levels of *slc44a2*, *chdh*, *ck,* and *chpt1* in the 0.80% choline group were significantly higher than those in the control group (Figure 3F).

### 3.4. Determination of Incubation Concentrations of Oleic Acid (OA) and Choline in Bighead Carp Hepatocytes

**Figure 4:**
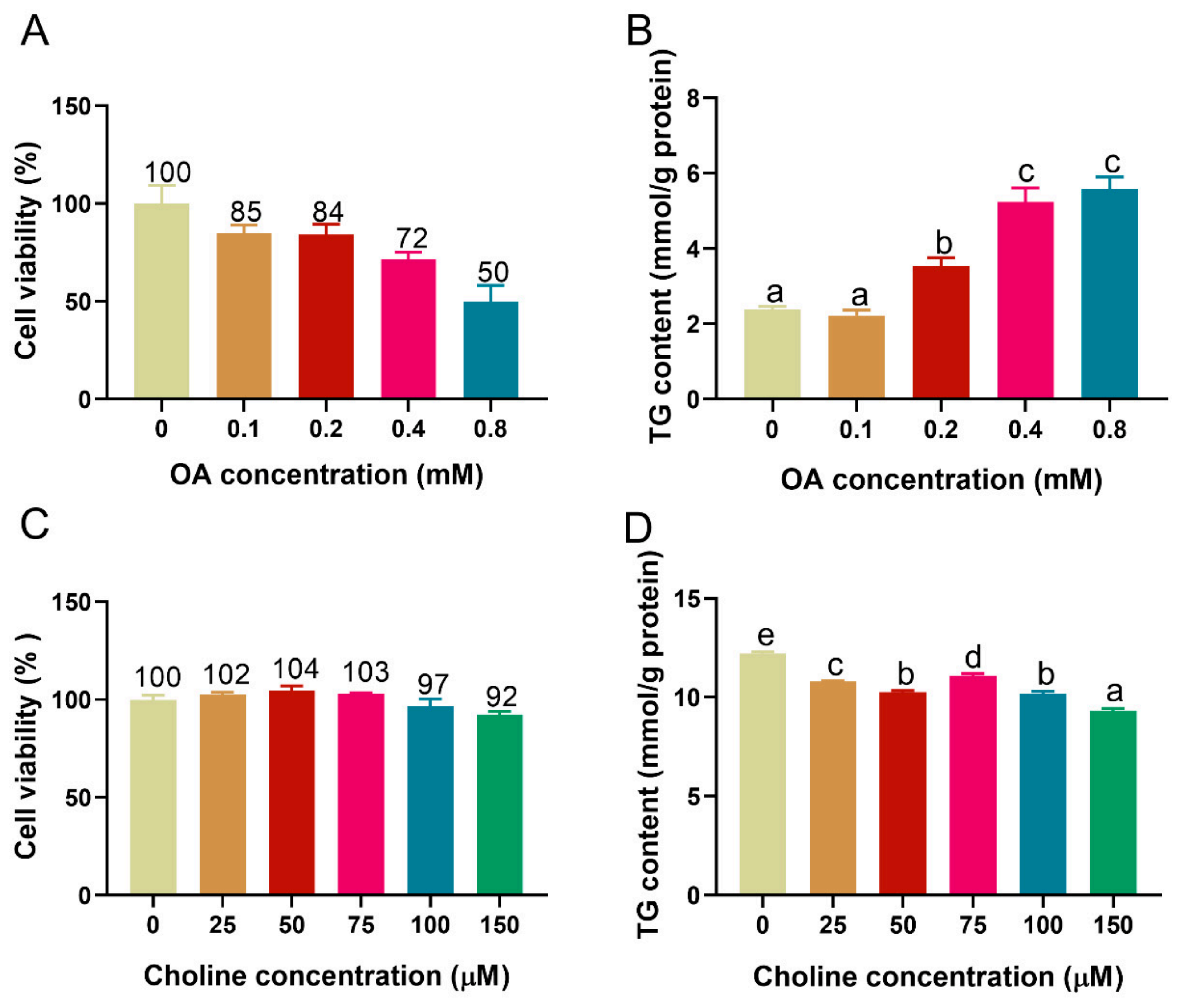
Determination of incubation concentrations of oleic acid (OA) and choline in bighead carp hepatocytes. (**A**): Effects of different oleic acid concentrations on the cell viability of primary hepatocytes from bighead carp; (**B**): Effects of different oleic acid concentrations on the TG content of primary hepatocytes from bighead carp; (**C**): Effects of different choline concentrations on the viability of OA-incubated primary hepatocytes from bighead carp; (**D**): Effects of different choline concentrations on the TG content of OA-incubated primary hepatocytes from bighead carp. Note: The numbers on the bars represent the mean values of cell viability; Data were expressed as means with SD (*n* = 3). Distinct superscripts above the bars indicate significant variations (*p* < 0.05).

Figure 4A reveals that the increase in OA concentration reduced cell viability. The concentrations of 0.2, 0.4, and 0.8 mmol/L OA significantly increased cellular TG content (Figure 4B). Based on the combined results of cell viability and TG level changes, 0.2 mmol/L was selected as the optimal modeling concentration. After choline supplementation, no significant difference in cell viability was observed among all groups (Figure 4C). The treatment of 50 μmol/L choline produced the most prominent improvement in cell viability and markedly reduced cellular TG content (Figure 4D). Accordingly, 50 μmol/L was determined as the optimal intervention concentration.

### 3.5. Effects of Choline Incubation on TG Content and FAS Activity in High-Lipid Hepatocytes of Bighead Carp

**Figure 5:**
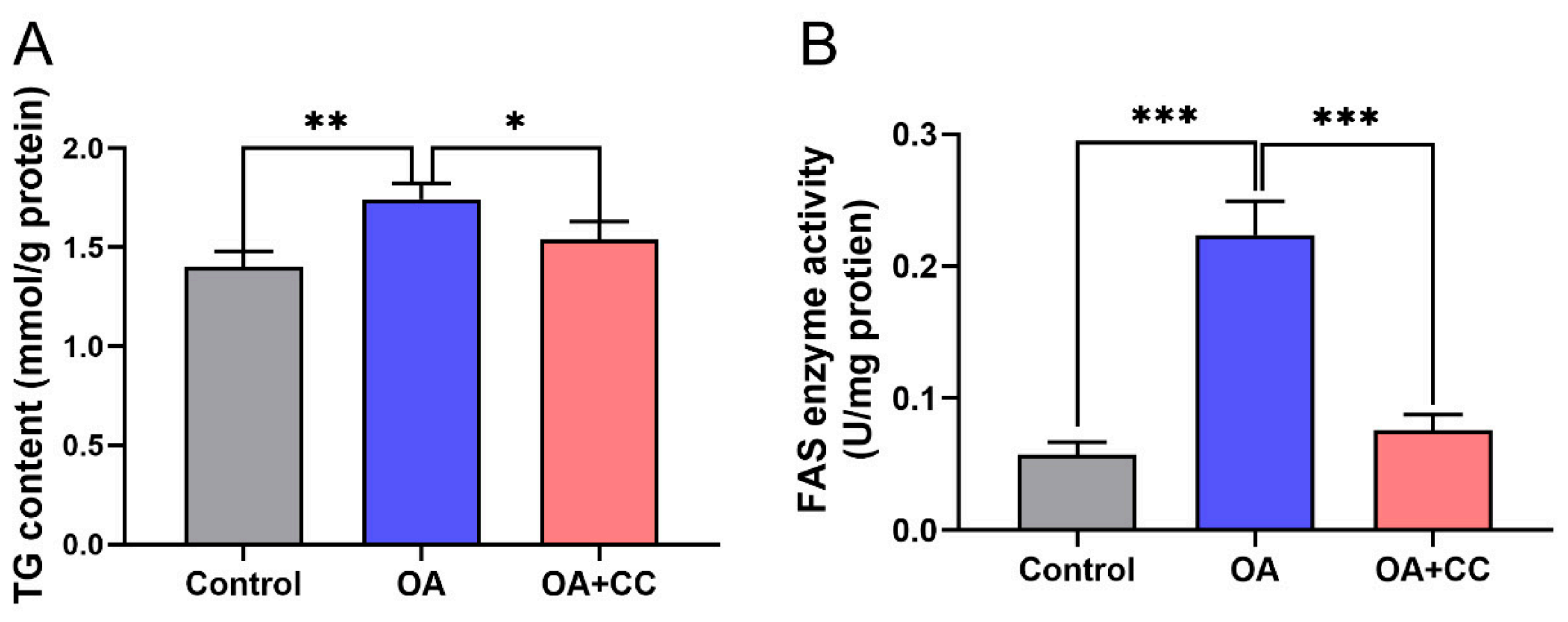
Effects of choline on TG content and FAS activity in high-lipid hepatocytes of bighead carp. (**A**): Cellular TG content; (**B**): Cellular FAS activity. Control: Control group; OA: High-lipid model group; OA + CC: High-lipid plus choline group. Note: * *p* < 0.05, ** *p* < 0.01, *** *p* < 0.001. Data were expressed as means with SD (*n* = 3).

Compared with the control group, the cellular TG content and FAS activity were significantly increased in the OA model group. In contrast, relative to the OA model group, the OA + CC group exhibited significant reductions in TG content and FAS activity (Figure 5).

### 3.6. Effects of Choline Incubation on Gene Expression in High-Lipid Hepatocytes of Bighead Carp

**Figure 6:**
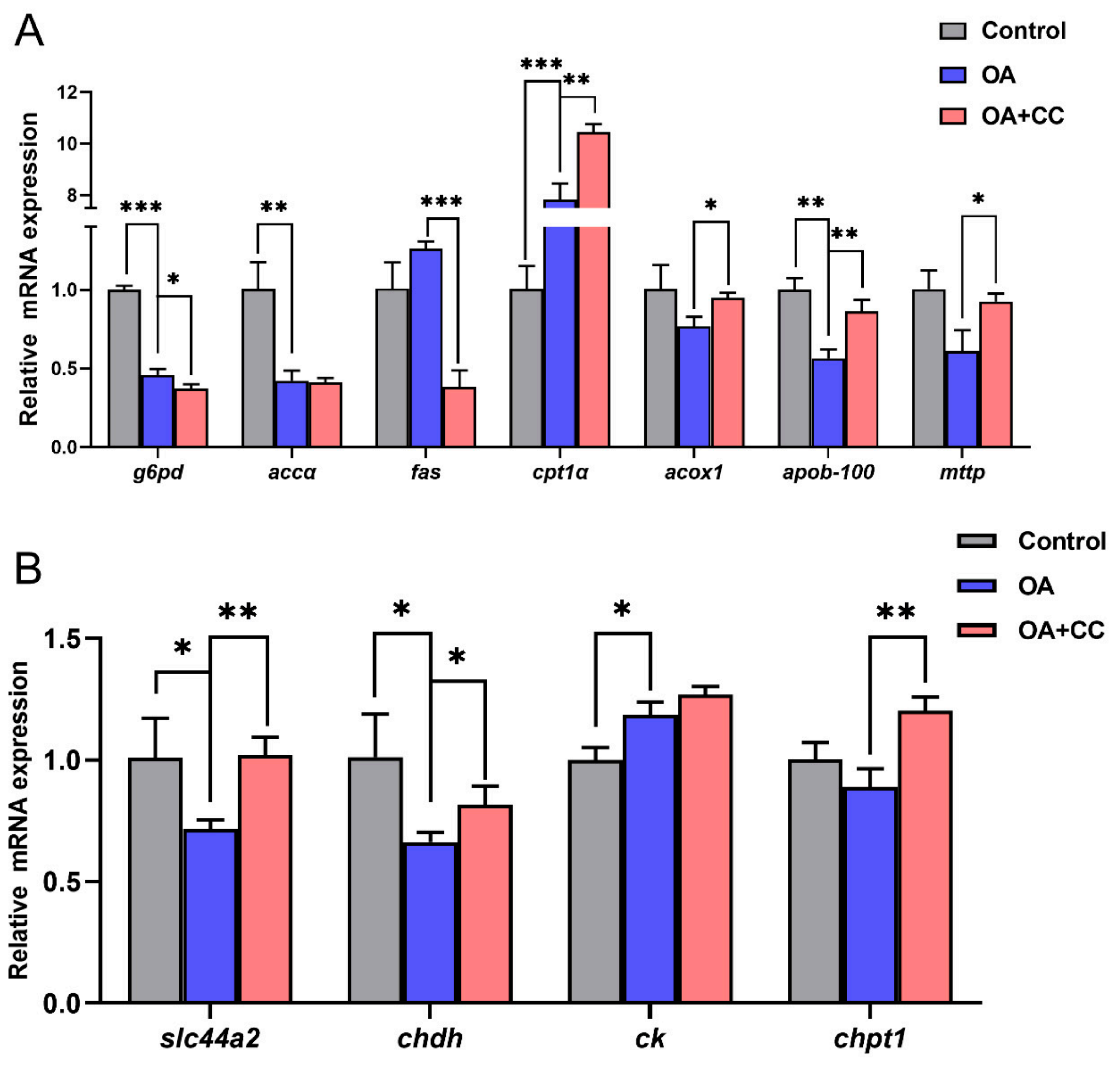
Effects of choline incubation on gene expression in high-lipid hepatocytes of bighead carp. (**A**): Lipid metabolism-related genes; (**B**): Choline metabolism-related genes. Note: * *p* < 0.05, ** *p* < 0.01, *** *p* < 0.001. Data were expressed as means with SD (*n* = 3).

Compared with the control group, OA treatment significantly upregulated the mRNA expression level of cpt1α, while markedly downregulating the expression of *g6pd*, *accα*, *apob-100* and *mttp*. No significant difference was observed in the expression of *fas* and *acox1*. Compared with the OA group, OA + CC treatment significantly downregulated the expression levels of *g6pd*, *acca* and *fas*, but significantly upregulated the expression of *cpt1α*, *acox1*, *apob-100* and *mttp* (Figure 6A).

Compared with the control group, OA treatment significantly downregulated the mRNA levels of *slc44a2* and *chdh*, while exerting no significant effect on the mRNA expression of *chpt1*. Compared with the OA group, OA + CC treatment markedly upregulated the expression levels of *slc44a2*, *chdh* and *chpt1*.

## 4. Discussion

In this study, an in vivo feeding trial, an in vitro primary hepatocyte experiment, and untargeted lipidomics were combined for integrated analysis. The molecular mechanism by which choline chloride alleviates excessive hepatic lipid deposition in bighead carp was systematically elucidated. For the in vivo feeding trial, histological observation and lipid deposition analysis of the liver showed that dietary choline deficiency induced obvious hepatocyte vacuolization and nuclear displacement in bighead carp. Massive lipid droplet accumulation was observed via Oil Red O staining, accompanied by a significant increase in hepatic TG content. These findings are consistent with previous reports on choline deficiency-induced fatty liver in fish species including hybrid yellow catfish (*Pelteobagrus fulvidraco*) [36], Yellowtail kingfish (*Seriola lalandi*) [37], and Largemouth bass (*Micropterus salmoides*) [38]. With the increase in dietary choline level, the number of hepatic lipid droplets and hepatic TG content decreased in a dose-dependent gradient. The 0.80% and 1.00% supplementation groups exhibited the most pronounced ameliorative effects, indicating that dietary choline supplementation at 0.80% and 1.00% could effectively maintain the morphological integrity of hepatocytes and reduce ectopic lipid deposition in the liver. The study reported that dietary choline supplementation contributed to the reduction in hepatic lipid droplet deposition in yellow catfish [36]. Similar results have also been documented in blunt snout bream (*Megalobrama amblycephala*) [11], black seabream (*Acanthopagrus schlegelii*) [6], and hybrid grouper [7]. Studies have demonstrated that FAS acts as a crucial regulatory enzyme involved in fatty acid biosynthesis and plays a pivotal role in maintaining lipid homeostasis in fish and aquatic animals [39]. At the cellular level, we also found that 50 μM choline significantly reversed oleic acid-induced TG accumulation and the elevation of FAS activity in bighead carp hepatocytes. This result confirms at the cellular level that 50 μM choline can effectively alleviate oleic acid-induced excessive TG accumulation in bighead carp hepatocytes by inhibiting the activity of FAS, a key rate-limiting enzyme for fatty acid biosynthesis, and exerts a marked ameliorative effect on hepatic lipid deposition. Replacing fish oil with vegetable oil in feed can significantly reduce production costs. However, studies have shown that vegetable oils are deficient in n-3 long-chain polyunsaturated fatty acids (n-3 LC-PUFA), especially eicosapentaenoic acid (EPA) and docosahexaenoic acid (DHA). Substitution of fish oil with vegetable oil tends to induce hepatic steatosis in fish [40]. The lipid sources of experimental diets in this study were derived from soybean oil and corn oil, which may partially explain why a high supplemental level of choline was required to reduce lipid accumulation in liver tissues.

Hepatic lipid deposition in fish is regulated by the expression of lipid metabolism-related genes [41]. In the present study, both in vivo and in vitro assays showed that compared with the control group, choline supplementation significantly downregulated the mRNA expression levels of key lipogenic genes including *g6pd*, *accα* and *fas* in the liver, while markedly upregulating the mRNA expression of fatty acid β-oxidation-related genes such as *cpt1α* and *acox1*. The study also reported that dietary supplementation with 0.3% choline reduced the expression levels of hepatic *fas*, *g6pd* and *acc* in largemouth bass relative to the control group [42]. Dietary choline supplementation also downregulates the expression levels of hepatic *accα* and *fas* in black seabream and hybrid grouper [6,7]. Furthermore, at the cellular level, 50 μM choline significantly upregulated the expression of *apob-100* and *mttp*, suggesting that choline may accelerate the hepatic outward transport of TG by enhancing the assembly and secretion capacity of VLDL, thereby reducing hepatic lipid deposition. The study also found that dietary supplementation with 1800 mg/kg choline markedly upregulated the expression levels of *apob-100* and *mttp* in blunt snout bream, and further induced the synthesis and assembly of VLDL [11]. Dietary supplementation with choline at 5 g/kg and 10 g/kg significantly upregulated the *apob-100* gene in the liver of hybrid grouper [7]. This suggests that choline can effectively mitigate hepatic lipid accumulation by inhibiting hepatic de novo lipogenesis, accelerating fatty acid β-oxidation, and facilitating the efflux of hepatic triglycerides. To further elucidate how choline deficiency affects lipid metabolism in bighead carp, we compared the differences in hepatic lipid metabolites between the 0.00% and 0.80% choline supplementation groups. It was found that compared with the 0.00% choline group, the levels of hepatic PC, PE, and PS were significantly upregulated in the 0.80% choline supplementation group, and glycerophospholipid metabolism was identified as the core enriched pathway. PC serves as the key structural component of VLDL, and its elevation can directly improve the efficiency of hepatic lipid export [43]. PE and PS are involved in the regulation of cell membrane homeostasis and lipid droplet formation. Their increased levels can reduce the accumulation of intracellular lipid droplets in hepatocytes and enhance the structural stability of the hepatic cell membrane [44,45]. Collectively, choline maintains hepatic lipid homeostasis through dual pathways: inhibiting endogenous hepatic lipogenesis and accelerating lipid export from the liver.

Choline metabolism in vivo mainly involves choline transport, choline oxidation, and choline phosphorylation pathways. Solute carrier family 44 member A2 (SLC44A2) is a choline transporter located on the cell membrane, which is primarily responsible for the transmembrane transport of choline [46]. Choline dehydrogenase (CHDH) participates in the oxidation process of choline. It first catalyzes the oxidation of choline to betaine, provides methyl groups for carnitine synthesis, and further enhances the *cpt1α*-mediated mitochondrial transport of fatty acids [47]. Choline kinase (CK) and choline phosphotransferase (CHPT1) are key enzymes in the choline phosphorylation pathway, which catalyze the phosphorylation of choline to produce phosphatidylcholine (PC) [5,48,49]. In the present study, compared with the control group, the gene expression levels of *slc44a2*, *chdh*, *ck,* and *chpt1* were significantly upregulated in the choline supplementation group in both in vivo and in vitro hepatocyte experiments. This indicates that dietary choline supplementation can markedly enhance the transport, oxidation, and phosphorylation of choline in the liver, which is likely an important mechanism underlying the upregulation of *cpt1* gene expression and the increase in PC content induced by choline. Similarly, the study also reported that dietary choline supplementation significantly upregulated the gene expression of *slc44a2*, *ck,* and *chpt1* in the hepatopancreas [8].

**Figure 7:**
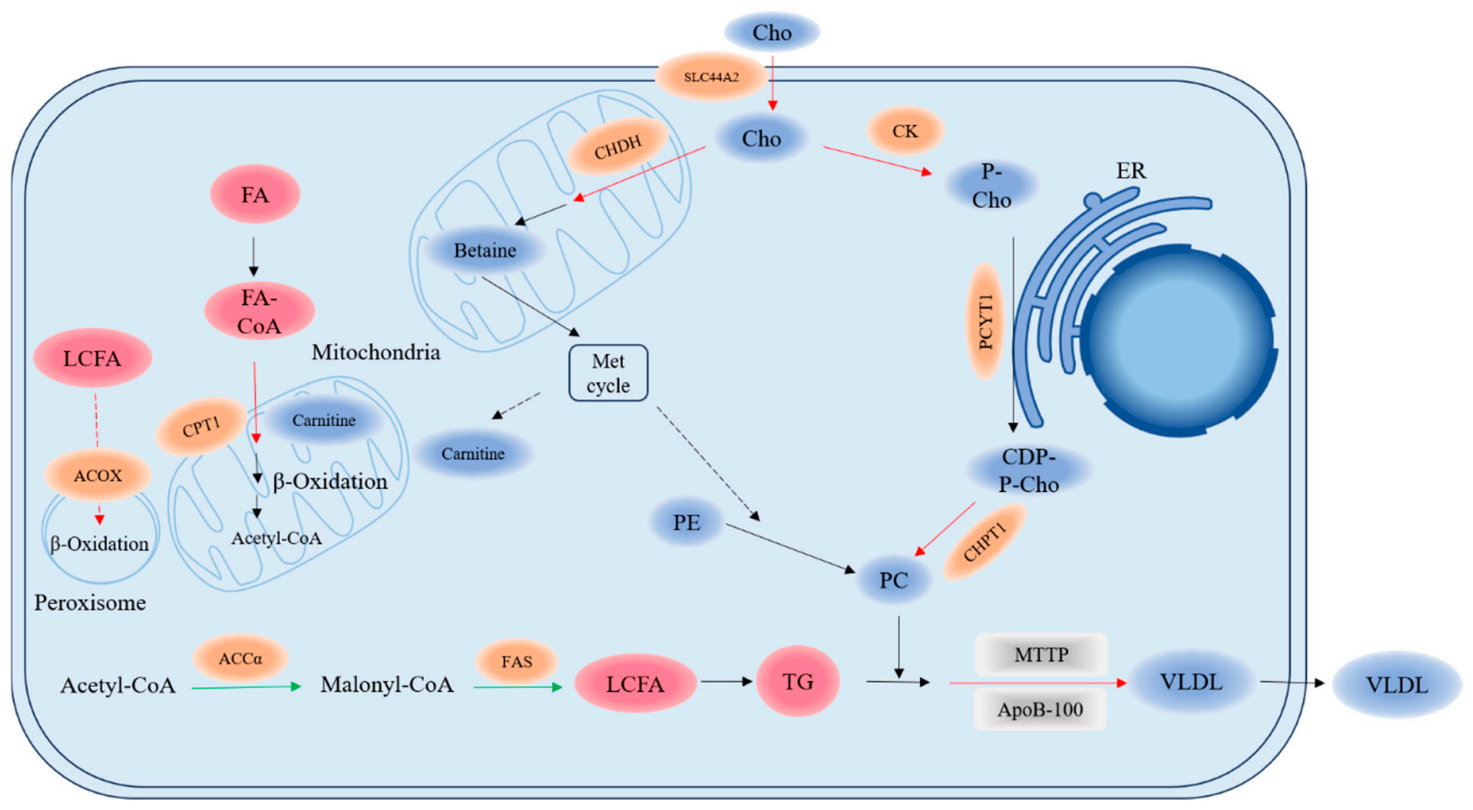
Predicted molecular mechanism of choline regulating lipid metabolism in hepatocytes of bighead carp. Note: Red arrow: upregulation; Green arrow: downregulation; Dashed arrow: multi-step reaction. Cho: Choline, P-Cho: Phosphocholine, CDP-P-Cho: Cytidine diphosphate choline, PC: Phosphatidylcholine, PE: Phosphatidylethanolamine, FA: Fatty acid, LCFA: Long-chain fatty acid, TG: Triglyceride, VLDL: Very low density lipoprotein, SLC44A2: Solute carrier family 44 member A2, CHDH: Choline dehydrogenase, CK: Choline kinase, CHPT1: Choline phosphotransferase, CPT1α: Carnitine palmitoyltransferase 1α, ACOX: Acyl-CoA oxidase, ACCα: Acetyl-CoA carboxylase α, FAS: Fatty acid synthase, MTTP: Microsomal triglyceride transfer protein, ApoB-100: Apolipoprotein B-100.

Based on the present data, we hypothesize the mechanism by which choline alleviates hepatic lipid deposition in bighead carp (Figure 7). After choline supplementation, choline uptake is increased in hepatocytes of bighead carp. Once transported into hepatocytes, choline may upregulate the activities of CK and CHPT1, thereby elevating PC content. The resultant PC can assemble with MTTP and ApoB-100 to form VLDL, which may facilitate the efflux of TG from cells. On the other hand, choline may upregulate CHDH to promote the oxidation of choline into betaine, which enhances methyl group supply and elevates carnitine levels. The increased carnitine may assist CPT1 in transporting lipids into mitochondria, presumably strengthening mitochondrial lipid β-oxidation. Meanwhile, ACOX may also be upregulated to facilitate lipid β-oxidation in peroxisomes. In addition, choline supplementation likely downregulates the activities of ACC and FAS, thereby suppressing lipid synthesis.

## 5. Conclusions

This study confirmed that dietary supplementation with 0.80% choline chloride could maintain the structural integrity of the liver and effectively alleviate hepatic lipid deposition in bighead carp. This optimal supplementation level has high practical application value for preventing and treating fatty liver disease and improving overall aquaculture benefits in fish farming. Nevertheless, this study has several limitations. First, all analyses were conducted only at the transcriptional level without protein validation, and results are species-specific to bighead carp. Second, only exogenous choline chloride was supplemented, while the total endogenous choline concentration of experimental diets was not quantified. Even so, the obtained results can still provide a reference for subsequent research and practical production. Choline alleviates hepatic lipid deposition and improves liver health mainly through a dual regulatory mechanism: modulating the glycerophospholipid metabolism pathway and regulating its own transport (*slc44a2*), oxidation (*chdh*), and phosphorylation (*ck*, *chpt1*). Meanwhile, choline inhibits lipogenesis-related gene (*fas*) expression, promotes fatty acid β-oxidation via upregulating (*cpt1α*), and facilitates hepatic TG export by regulating *apob-100* and *mttp*. The findings of this study provide a scientific basis for the nutritional prevention and control of fatty liver and the precise feed formulation of bighead carp.

## Figures and Tables

**Table 1:** Composition and nutrient levels of diets (dry basis %).

Item	Dietary Choline Chloride Added Levels (g/kg)					
	0.00	2.00	4.00	6.00	8.00	10.00
Ingredients (%)						
Fish meal	10.00	10.00	10.00	10.00	10.00	10.00
Soybean meal (Solvent-extracted soybean meal)	36.50	36.50	36.50	36.50	36.50	36.50
Flour	22.00	22.00	22.00	22.00	22.00	22.00
Cotton meal (Expeller cottonseed meal)	20.00	20.00	20.00	20.00	20.00	20.00
Soybean oil	3.00	3.00	3.00	3.00	3.00	3.00
Corn oil	3.00	3.00	3.00	3.00	3.00	3.00
^1^ Mineral premix	1.00	1.00	1.00	1.00	1.00	1.00
^2^ Vitamin premix	1.00	1.00	1.00	1.00	1.00	1.00
Calcium dihydrogen phosphate	2.50	2.50	2.50	2.50	2.50	2.50
Cellulose powder	1.00	0.80	0.60	0.40	0.20	0.00
Choline chloride (50%)	0.00	0.20	0.40	0.60	0.80	1.00
Proximate composition (%)						
Moisture	8.56	7.48	7.57	7.37	8.63	8.41
Crude protein	43.57	43.02	43.08	43.21	43.49	43.48
Crude lipid	6.61	7.18	7.21	7.01	7.17	7.17
Crude ash	9.37	9.53	9.43	9.59	9.50	9.67

^1^ Mineral premix (g/kg): MgSO_4_, 20 g; FeSO_4_·7H_2_O, 20 g; MnSO_4_·H_2_O, 2 g; CuSO_4_·5H_2_O, 4 g; Kal (SO_4_)_2_·12H_2_O, 6 g; ZnSO_4_·7H_2_O, 54 g; KCl, 64 g; CoCl_2_·6H_2_O, 0.4 g; KI, 0.6 g. ^2^ Vitamin premix (g/kg): VA, 1.47 g; VD_3_, 0.022 g; VK, 8.9 g; VE, 44.5 g; VB_1_, 17.8 g; VB_2_, 17.8 g; VB_6_, 17.8 g; VB_12_, 0.018 g; biotin, 0.089 g; folicacid, 4.45 g; niacin, 89.12 g; Ca-D-pantothenate, 44.5 g; inositol, 89.12 g; cellulose, 575.05 g. Dietary choline chloride was supplemented at 0.00, 2.00, 4.00, 6.00, 8.00 and 10.00 g/kg diet, equivalent to 0.00%, 0.20%, 0.40%, 0.60%, 0.80% and 1.00%. The supplemental choline chloride is a commercial product with 50% active ingredient and corn bran as a carrier. All addition levels in this table are calculated based on the commercial product.

**Table 2:** Primers used for real-time quantitative PCR analysis.

Gene Name	Forward Primer (5′–3′)	Reverse Primer (5′–3′)	Gene Bank
*g6pd*	AAGCTGCCTGATGCTTATGA	TCGGCTCCCGTATTTGTAT	OR820521
*fas*	AGGCAGACACTAGAGAGGGA	CATCCGGCTGAGATCTGACT	OR820520
*accα*	GAGTGCAGGTACAGTGGAGT	TGCAACCCGTACATCACTCT	OR820516
*cpt1α*	AGCAGCAGATGGAGAGGATC	TGCTCTGTGTCATCCAGTGT	OR820519
*acox1*	ACCACTGCTACTTACGACCC	TGAGCCAGGACTATAGCGTG	OR820517
*apob-100*	ATGAGACCTTCCATCCTGCC	CGAGGCATTCTTGAACTCCG	OR820518
*mttp*	CTGCCGCATGAGATGAACAA	AGTTTGTGCCATGAAGCCTG	OR820525
*slc44a2*	TCCACATCCAATGAGCCAGT	CAGCATCAGGACATTCGGTG	OR820533
*ck*	TTGCTATCCTGGCTGAGAGG	CGTGAAACCTGGCGATCTTT	OR820527
*chpt1*	GGCGGGTTCCTGTATTCAAC	TGAGGATGAGGCCAATGTGT	OR820528
*chdh*	AACCTCTGCGACGACAAGTA	GATCGTAGTCCCAGCCTTCA	OR820526
*gapdh*	GGTGACCCGTGCTGCTTT	GCCTTAACCTCGCCCTTGT	OR820522
*β-actin*	GAGGCTACTCTTTCGTTACAACC	CGGGCAACTCGTAGCTCTT	AY170122.2

*g6pd*, glucose-6-phosphate dehydrogenase; *fas*, fatty acid synthase; *accα*, acetyl-CoA carboxylase alpha; *cpt1α*, Carnitine palmitoyltransferase 1 alpha; *acox1*, acyl-CoA oxidase; *apob-100*, apolipoprotein B-100; *mttp*, microsomal triglyceride transfer protein; *slc44a2*, solute carrier family 44 member A2; *ck*, Choline kinase; *chpt1*, choline phosphotransferase; *chdh*, Choline dehydrogenase; *gapdh*, Glyceraldehyde-3-phosphate dehydrogenase; *β-actin* Beta-actin.

## Data Availability

The original contributions presented in this study are included in the article. Further inquiries can be directed to the corresponding author.
